# The Effect of Silicon on Photosynthesis and Expression of Its Relevant Genes in Rice (*Oryza sativa* L.) under High-Zinc Stress

**DOI:** 10.1371/journal.pone.0113782

**Published:** 2014-11-26

**Authors:** Alin Song, Ping Li, Fenliang Fan, Zhaojun Li, Yongchao Liang

**Affiliations:** 1 Ministry of Agriculture Key Laboratory of Crop Nutrition and Fertilization, Institute of Agricultural Resources and Regional Planning, Chinese Academy of Agricultural Sciences, Beijing, China; 2 Ministry of Education Key Laboratory of Environment Remediation and Ecological Health, College of Environmental & Resource Sciences, Zhejiang University, Hangzhou, China; Universidade Federal de Vicosa, Brazil

## Abstract

The main objectives of this study were to elucidate the roles of silicon (Si) in alleviating the effects of 2 mM zinc (high Zn) stress on photosynthesis and its related gene expression levels in leaves of rice (*Oryza sativa* L.) grown hydroponically with high-Zn stress. The results showed that photosynthetic parameters, including net photosynthetic rate, transpiration rate, stomatal conductance, intercellular CO_2_ concentration, chlorophyll concentration and the chlorophyll fluorescence, were decreased in rice exposed to high-Zn treatment. The leaf chloroplast structure was disordered under high-Zn stress, including uneven swelling, disintegrated and missing thylakoid membranes, and decreased starch granule size and number, which, however, were all counteracted by the addition of 1.5 mM Si. Furthermore, the expression levels of *Os08g02630* (*PsbY*), *Os05g48630 (PsaH)*, *Os07g37030* (*PetC), Os03g57120 (PetH)*, *Os09g26810* and *Os04g38410* decreased in Si-deprived plants under high-Zn stress. Nevertheless, the addition of 1.5 mM Si increased the expression levels of these genes in plants under high-Zn stress at 72 h, and the expression levels were higher in Si-treated plants than in Si-deprived plants. Therefore, we conclude that Si alleviates the Zn-induced damage to photosynthesis in rice. The decline of photosynthesis in Zn-stressed rice was attributed to stomatal limitation, and Si activated and regulated some photosynthesis-related genes in response to high-Zn stress, consequently increasing photosynthesis.

## Introduction

Zinc (Zn) is the second most abundant transitional metal after iron [1]. Under natural conditions, Zn is present in soils at low concentrations, ranging from 10 to 300 mg kg^−1^, with an average of approximately 50 mg kg^−1^ [2]. At these concentrations, Zn is an essential element for plant metabolism and growth. In most crops, the typical leaf Zn concentration ([Zn]_leaf_) required for adequate growth approximates 15–20 mg Zn kg^−1^ DW [1]. However, human activities, such as mining operations, have enhanced the Zn levels in numerous contaminated sites to concentrations that are potentially harmful to the environment and to human health [3], [4]. Among the heavy metals, Zn is one of the essential micro-minerals for normal growth of plants [5] and is involved in various biological activities, functioning as a cofactor within plant cells in a variety of physiological processes, including electron transfer in photosynthesis, mitochondrial respiration, superoxide scavenging, lignification of cell walls, and ethylene sensing [1], [6], [7], [8]. Depending on the dose and the chemical form of Zn, it can act as a nutrient, an antioxidant, or even a toxin [9]. A Zn deficiency can decrease the chlorophyll content and photosynthetic rate, change the activity of superoxide dismutase and inhibit plant growth [10], [11]. However, if the Zn concentration is too high, Zn can become highly toxic to plant and damage the plant's metabolism [12]. At the organismal level, excess Zn inhibits seed germination, plant growth and root development, and causes leaf chlorosis [13]. Chlorosis may arise partly from an induced iron (Fe) deficiency as hydrated Zn^2+^ and Fe^2+^ ions have similar radii. As a result, photosynthesis is inhibited under high-Zn stress. At the molecular level, excess Zn can alter gene expression. van de Mortel et al [14] reported many differentially regulated genes that were identified using genechips from *Arabidopsis thaliana* and *Thlaspi caerulescens* plants exposed to excess Zn. These were genes involved in various biological processes (e.g. lignin biosynthesis), including genes encoding defense proteins associated with oxidative stress. Recent studies [15], [16] have demonstrated the effect of Zn stress on the photosynthetic rate, chlorophyll content and chloroplast ultrastructure in plants. The data suggest there is crosstalk between Zn-induced differentially expressed genes and photosynthetic genes, representing a complex mechanism developed to cope with Zn toxicity. However, the majority of the differentially regulated genes have not been functionally identified. Additionally, the biochemical mechanisms for Zn toxicity and the adaptation mechanisms of plants under Zn stress are not well understood.

Silicon (Si) is the second most abundant element in soil. It is present as silicic acid in the soil solution at concentrations normally ranging from 0.1 to 2.0 mM, roughly two orders of magnitude higher than the concentrations of phosphorus in soil solutions [17], [18], [19]. Silicon concentrations vary greatly in plant aboveground parts, ranging from 0.1 to 10.0% SiO_2_ of dry weight or even higher [19], [20], [21]. Although Si has not been considered an essential element for higher plants, it is beneficial for the healthy growth and development of many plant species, particularly for rice which contains about 10% SiO_2_ in shoots on a dry weight basis [22], [23], [24], [25]. The beneficial effects of Si are particularly distinct in plants exposed to abiotic or biotic stress [24], [26], [27]. There is an increasing body of literature showing that the application of Si enhances the tolerance of some plant species to toxic metals, including manganese (Mn) [28], [29], aluminum (Al) [30], [31], cadmium (Cd) [32], [33], [34] and arsenic (As) [35], [36], [37], [38], [39]. Similarly, Si has been described as an effective remedy for Zn toxicity in many plants, such as maize and rice [40], [41], [42]. In the past, it has been determined that excess Zn could inhibit photosynthesis in *Arabidopsis*, beans and pakchoi [43], [44], [45], but there have been no reports on the effects of Si on photosynthesis under excess Zn. Previous studies have shown that Si alleviates growth inhibition and oxidative damage [42]. Therefore, it is hypothesized that Si may influence the photosynthetic capacity and expression levels of related genes. In the present study, the effects of Si on photosynthesis, chloroplast structure and chlorophyll parameters, as well as the expression of the genes responsible for photosynthesis, were investigated in rice exposed to high-Zn stress.

## Materials and Methods

### Plant material and growth conditions

#### Experiment A

The hydroponics experiments were carried out in an environmentally-controlled growth chamber from March to May in 2013. To screen out a Zn-sensitive variety, seedlings of 20 rice varieties were grown hydroponically with different Zn levels (0.15 µM (control), 0.04, 0.08, 0.16, 0.8, 2.0 and 4.0 mM) [42]. The Zn tolerance was assessed as a tolerant index (TI) by calculating root length according to the formula, TI  =  Root length (treated)/Root length (control) [43]. The root and shoot dry weights were determined after 7-day treatment. Compared with the control, the treatment with 2.0 mM Zn in the culture solution decreased root growth by about 50% in cultivar FYY-326 on which toxic symptoms were visible after treatment for one week [42]. According to Hoffmann and Schenk [46] the 2.0 mM Zn concentration was selected as toxicity threshold which led to a growth reduction by 50% (EC_50_). Based on our previous screening studies [42], a Zn-sensitive rice (*Oryza sativa* L.) cultivar, FYY-326, was used in this study. Rice seeds were surface-sterilized with 30% H_2_O_2_ (10%) for 30 min, followed by rinsing thoroughly with distilled water and germinated on moist filter paper for 48 h in an incubator at 35°C. After burgeoned, seeds were sown in the plastic containers filled with quartz sand and irrigated with 1/2-strength Kimura B nutrient solution [20]. The basic nutrient solution was prepared with de-ionized water containing 0.41 µmol L^−1^ Si. The composition of the basic nutrient solution was: (NH_4_)_2_SO_4_ 0.37 mM, MgSO_4_·7H_2_O 0.55 mM, KNO_3_ 0.18 mM, Ca(NO_3_)_2_·4H_2_O 0.37 mM, KH_2_PO_4_ 0.21 mM, NaEDTAFe·H_2_O 20 µM, MnCl_2_·4H_2_O 6.7 µM, (NH_4_)_6_Mo_7_O_4_·4H_2_O 0.015 µM, ZnSO_4_·7H_2_O 0.15 µM, CuSO_4_·5H_2_O 0.16 µM, and HBO_3_ 9.4 µM. The solution pH was adjusted to 5.4–5.6 with HCl or NaOH daily. Uniform 7-day-old plants were transferred to 5-L plastic pots (60 plants per pot). Plants were grown under controlled environmental conditions in a growth chamber with light/dark regime of 13/11 h, temperature regime of 35/25°C, photosynthetic photon flux density of 400 µmol m^−2^ s^−1^, and relative humidity of about 70%.

According to the previous reports [27], [47], [48], [49], K_m_ of silicic acid uptake is 0.15–0.35 mM, while V_max_ is reached at about 1.5–1.6 mM. Thus, in the present study, 1.5 mM silicic acid was applied. Plants were exposed to two concentrations of Zn, 0.15 µM (control) and 2.0 mM (high) respectively without or with 1.5 mM Si, added as K_2_SiO_3_·nH_2_O to the nutrient solution. Additional K introduced by K_2_SiO_3_·nH_2_O was subtracted from KNO_3_ and the resultant nitrate loss was supplemented with dilute nitric acid. The nutrient solutions were renewed completely every other day. In total, there were four treatments with each replicated 6 times. After having been treated for 72 h (3 days), plants of 3 replicates (pots) of each treatment were harvested for analysis of the gene expression by high-throughput sequencing and RT-PCR, and the remaining 3 replicates (pots) of each treatment were used for assay of Si and Zn concentrations.

#### Experiment B

This experiment was performed from May to July in 2013 and the experimental design and growth conditions were the same as described in Experiment A. There were four treatments with each replicated 6 times. After having been treated for 7 days, plants of 3 replicates (pots) of each treatment were used for determination of photosynthesis, fluorescence and chlorophyll parameters, and for examination of chloroplast ultrastructure, respectively, and the remaining 3 replicates (pots) of each treatment were used for measurement of biomass. The specific experimental design for both experiment A and B is listed in Table 1.

**Table 1:** Experimental design and measured index.

Treatments	Experiments	Treatment time (d)	Repetition	Measured index
Normal Zn-Si	A	3	3	Gene expression
Normal Zn+Si	A	3	3	Gene expression
High Zn-Si	A	3	3	Gene expression
High Zn+Si	A	3	3	Gene expression
Normal Zn-Si	A	3	3	Si and Zn concentrations
Normal Zn+Si	A	3	3	Si and Zn concentrations
High Zn-Si	A	3	3	Si and Zn concentrations
High Zn+Si	A	3	3	Si and Zn concentrations
Normal Zn-Si	B	7	3	Photosynthetic, fluorescence parameters, chlorophyll a and b and chloroplast ultrastructure
Normal Zn+Si	B	7	3	Photosynthetic, fluorescence parameters, chlorophyll a and b and chloroplast ultrastructure
High Zn-Si	B	7	3	Photosynthetic, fluorescence parameters, chlorophyll a and b and chloroplast ultrastructure
High Zn+Si	B	7	3	Photosynthetic, fluorescence parameters, chlorophyll a and b and chloroplast ultrastructure
Normal Zn-Si	B	7	3	Biomass
Normal Zn+Si	B	7	3	Biomass
High Zn-Si	B	7	3	Biomass
High Zn+Si	B	7	3	Biomass

### Determination of Zn and Si

The concentration of Zn and Si in shoot and root was determined according to the method of Song et al. [42].

### Assay of contents of chlorophyll a and b

Chlorophyll a and b contents were determined spectrophotometrically in an 80% acetone solution according to Arnon [50]. The pigment concentration of leaves was initially presented on a fresh weight basis. In order to avoid the complications of changing water concentration, the pigment concentration was also calculated on a dry weight basis according to water concentration of leaves.

### Chlorophyll fluorescence measurement

Chlorophyll fluorescence parameters were measured by PAM modulation chlorophyll fluorescence analyzer (Walz, Effeltrich, Germany). Prior to the measurements, the plant was dark adapted for 20 min. The minimal fluorescence (F_0_) with all PSII reaction centers (RCs) open was measured with modulated radiation low enough not to induce any photosynthesis in the leaf. The maximal fluorescence (F_m_) with all RCs closed was measured with a 0.8 s pulse of saturating radiation of 3000 µmol m^−2^ s^−1^ on dark-adapted leaves. Measurements were done between 09: 00 and 11: 00 am. The value of *F_v_* was calculated according to the formula: *F_v_* = *F_m_*−*F_0_*.

### Photosynthesis measurement

Net photosynthetic rate (*P*_n_), stomatal conductance (*G*_s_), transpiration rate (*T*_r_), intercellular CO_2_ concentration (*C_i_*) were measured four times before harvesting (seven days after Zn and/or Si treatments) using an open portable infrared gas analysis system (LI-6400, USA). Light intensity was 1000 µmol m^−2^ s^−1^; air temperature was 25°C; atmospheric CO_2_ concentration was about 430 µmol mol^−1^. Measurements were done on the second leaves of fully expanded from the top between 09: 00 and 11: 00 am.

### Electron microscopy (chloroplast ultrastructure)

Electron microscopic studies were made using the middle section of the 2^nd^ leaves of 7-day seedlings treated with Si and Zn. To prevent entry of air bubbles, the sections (1 mm) were cut with a sharp blade and immersed in 2.5% glutaraldehyde for 48 h, and were further immersed in 0.5% sodium sulfide solution (pH 7.2) for 30 min and subsequently rinsed with 0.1 M phosphate buffer, pH 7.2, for 3 times at 15-min intervals. Tissues were then postfixed in 1% OsO4 in 0.1 M phosphate buffer, pH 7.2, at 4°C for 12 h and dehydrated with a grade series of ethanol (30%-50%-70%- 85%-95%) and finally dehydrated with 100% ethanol 3 times at 15-min intervals. Samples were then embedded in LR white and polymerized at 60°C for 24 h.

For electron microscopy, ultrathin sections (70 nm in thickness) were cut with a diamond knife and placed on 200 mesh copper grids. The grids were stained with 2% uranyl acetate for 20 min followed by lead citrate for 5 min. The sections were then viewed on a Phillips EM 400 ST transmission electron microscope (TEM).

### Gene selection and quantitative real-time PCR analysis

After having grown for 72 h, plants were harvested and samples were collected to assay genes expression by fluorescent quantitative real-time reverse transcription-polymerase chain reaction.

**Figure 1:**
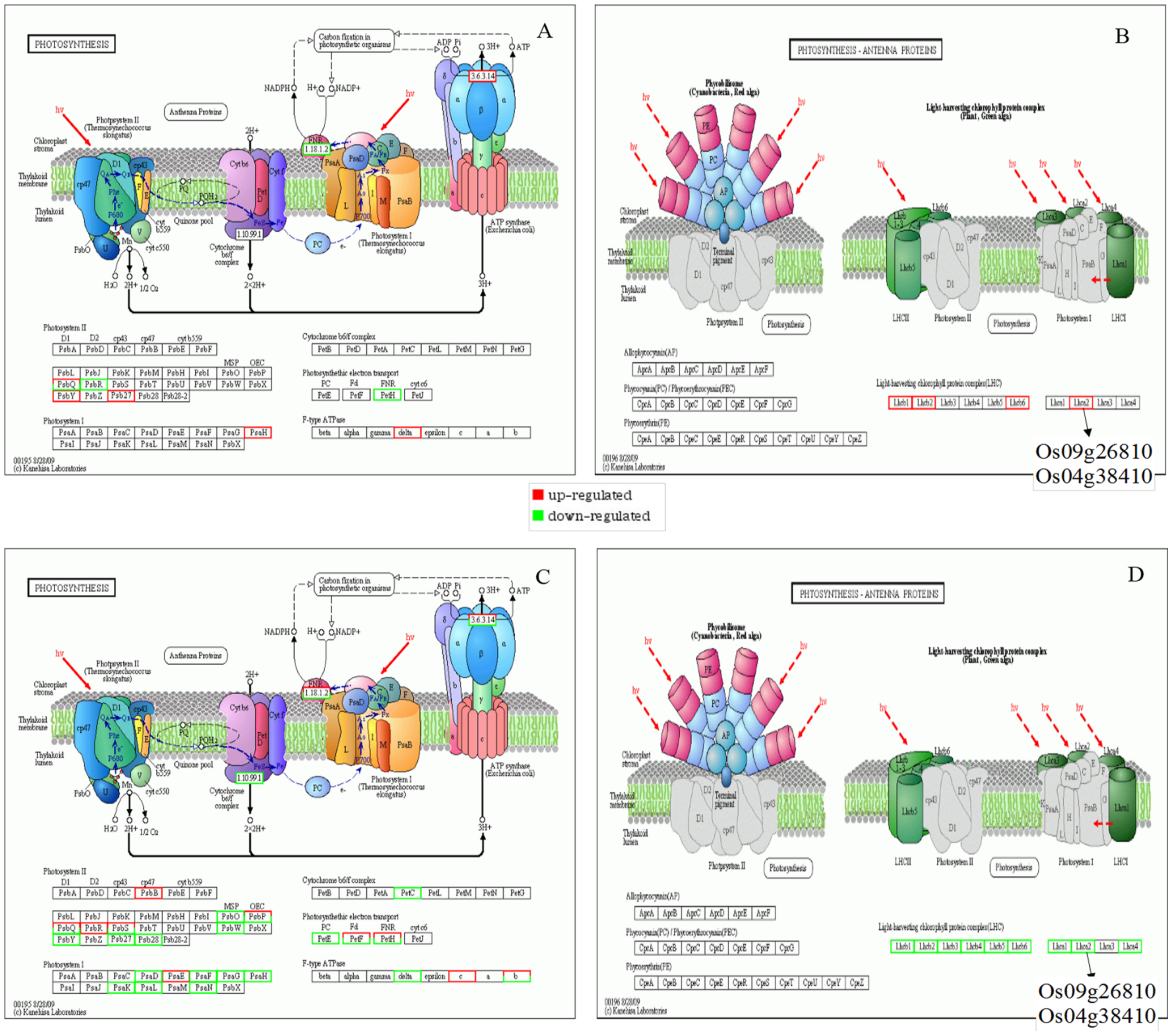
The pathway analysis of photosynthesis (A and C) and photosynthesis-antenna proteins (B and D) of Zn-sensitive rice (*Oryza sativa* L.) cultivar grown hydroponically with either normal (0.15 µM) or high (2 mM) Zn without or with 1.5 mM Si for 72 h through high-throughput sequencing.

Photosynthesis parameters and chloroplast ultrastructure were affected under high Zn stress, and which were alleviated with addition of Si. Differentially expressed genes were analyzed through high-throughput sequencing according to the results of physiological indicators. Based on the results of high-throughput sequencing (Figure 1), main genes related to photosynthesis were selected and further confirmed by real-time quantitative PCR. The high-throughput sequencing experimental process includes sample preparation and sequencing in Huada Genomics Company, Shenzhen, China. The main reagents and supplies are Illumina Gene Expression Sample Prep Kit and Solexa Sequencing Chip (flowcell), and the main instruments are Illumina Cluster Station and Illumina Genome Analyzer System. We identified differentially expressed genes between two samples referring to "The significance of digital gene expression profiles" [51]. To identify significantly differentially expressed genes, a combined criterion of FDR <0.001 and the absolute value of log_2_Ratio (Zn/CK) ≥ 1 in the Poisson distribution were adopted.

Gene specific primers (Table 2) **w**ere designed and synthesized by Baosheng Corporation, China. Total RNA isolated from rice tissues was first converted to cDNA using MMLV reverse transcriptase (Tiangen Corporation, China), according to the manufacturer's specifications, with random hexamer primers from Baosheng Corporation. The cDNA products generated were from three biological replicates for each sample. Reactions (20 µl) were performed in triplicate in 96-well plates (TempPlate Scientific, BIO-RAD, China). The program consisted of a Hot-Start activation step at 95°C for 10 s, followed by 40 cycles of: 95°C for 5 s, 60°C for 30 s. Amplification of the actin internal control was performed in the same 96-well plate as the other genes. Reactions were performed with the iCycler Real-Time PCR Detection System (Bio-Rad Laboratories INC., USA) employing the two-step amplification plus melting curve protocol. The values for threshold cycle (Ct) determination were generated automatically by iCycler software (Bio-Rad Laboratories). Relative quantitative method delta-delta C_T_ (2^−ΔΔC^_T_) [52] was used to describe expression patterns of selected genes by comparing the gene expression levels between Zn-stressed samples with or without Si and healthy samples.

**Table 2:** The conserved region of gene sequence and predicted product size for PCR used for detecting gene expression in the leaves of rice.

Gene name	Accession number	Primer name	Primer sequence (5′-3′)		PCR product Size (bp)
***Os08g02630***	*PsbY*	*AK060602*	F	CGCAGCGACATTGCACTCACC	104
			R	TGACGCCTTCTTCTTCCTCCTCTT	
***Os05g48630***	*PsaH*	*AK060254*	F	CTATCTACTCAGCTTTGCGTCCTCC	203
			R	CGCTCTTCTCGCCGTACTTGG	
***Os07g37030***	*PetC*	AK071634	F	GCACCACTTCGTCCTCAACCA	201
			R	TGTCAGGCAGCAAGTTCTTCACC	
***Os03g57120***	*PetH*	AK070598	F	CACTGGTGTTGCTCCATTCCG	255
			R	TCGCTGTACTCCTCGATCTTGTCCT	
***Os09g26810***	*Os09g26810*	AK067780	F	TCCCGGAGGTGCTGGAGAAAT	129
			R	CGTCCCAAACTCTAATCCCGTCAA	
***Os04g38410***	*Os04g38410*	AK066070	F	GTCTACATCCCGGACACCGACAA	292
			R	GCTACTGCTCCTCCGACCTCCAAT	

### Statistical analyses

All the experiment data presented in this paper were means of the data of two independent experiments and statistically examined by two-way analysis of variance. Statistical significance of the means of three replicates was compared at 0.05 probability level using Sigmastat for Windows Version 2.03 (SPSS Inc.) and all figures were drawn using SigmaPlot software (Version 12.5).

## Results

### Plant growth

**Figure 2:**
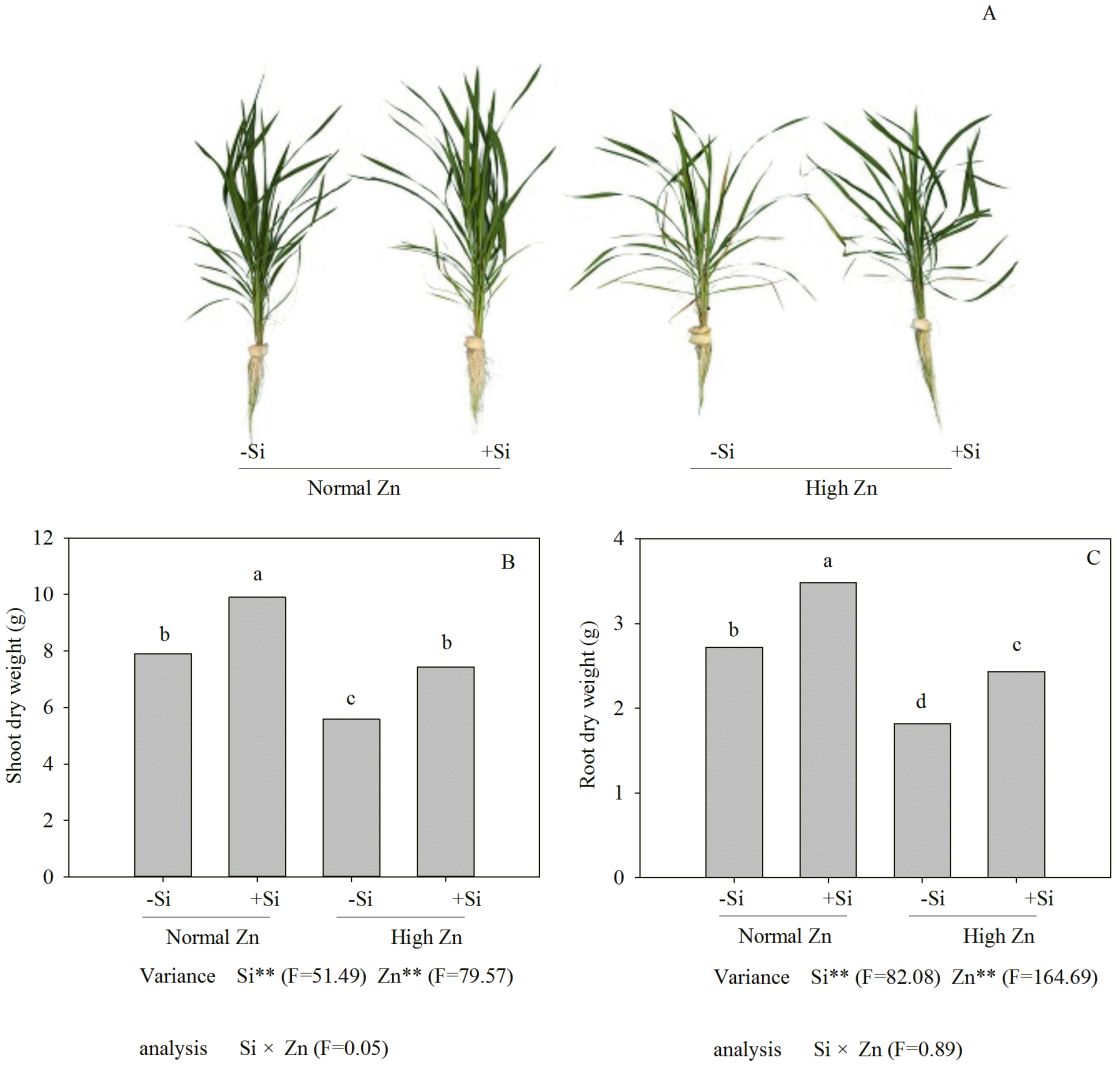
Zn toxicity symptoms (A), shoot (B) and root (C) dry weight of Zn-sensitive rice (*Oryza sativa* L.) cultivar grown hydroponically with either normal (0.15 µM) or high (2 mM) Zn without or with 1.5 mM Si for seven days. Data are means ± S.D. of three replicates. Note: P-values indicate significance level based on two-way ANOVA. ^*^P<0.05, ^**^P<0.01. Data followed by different letters are significantly different according to ANOVA (P <0.05).

A change was observed in plant biomass, in terms of shoot and root dry weight, as the Zn concentration increased compared with the control (Figure 2A). The shoot and root dry weights decreased by 29.4% and 33.2%, respectively, under high Zn stress compared with the control. However, the addition of 1.5 mM Si to high Zn treatments increased the shoot and root biomass compared with the corresponding Zn treatment alone (Figure 2B, C).

### Shoot and root Si and Zn concentrations

**Figure 3:**
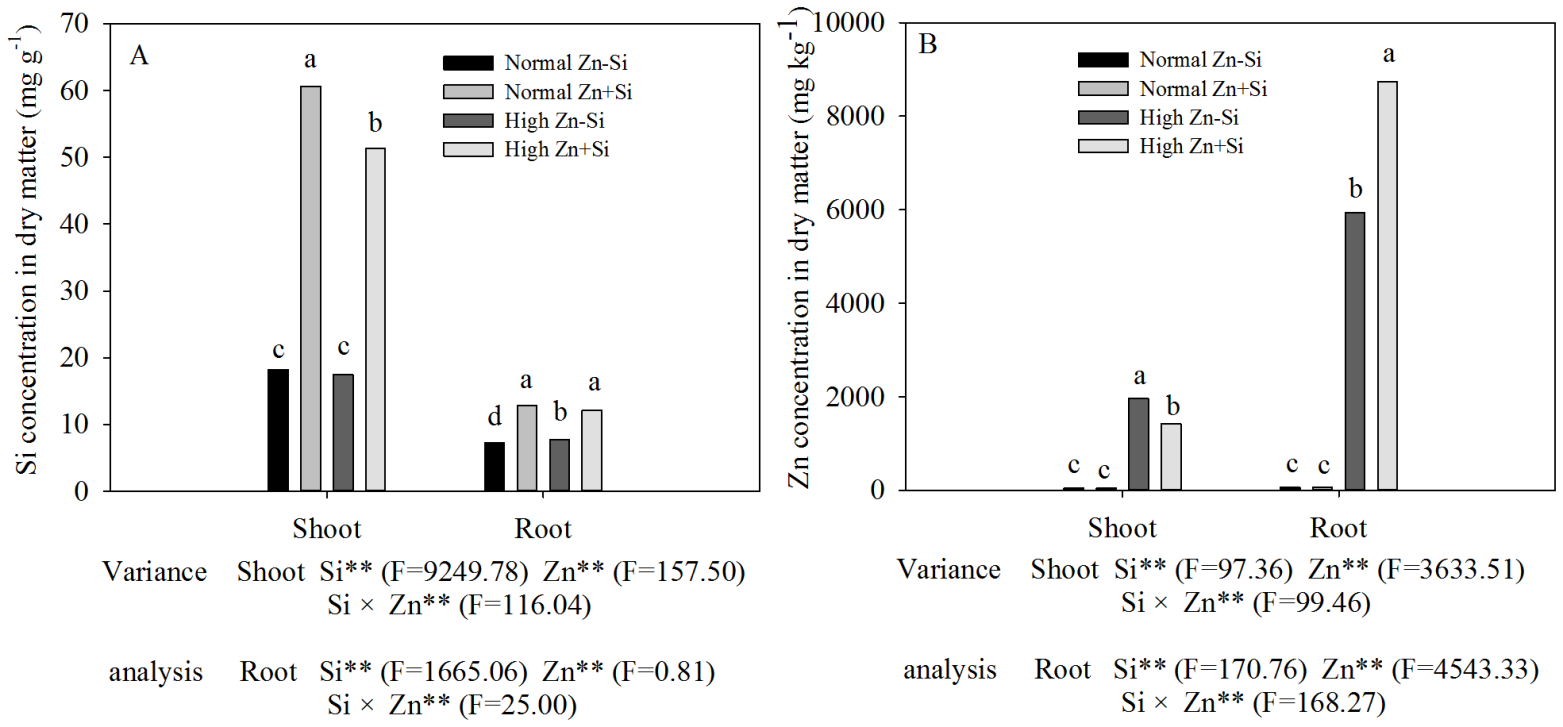
Si contents in dry matter of shoot and root (A) and Zn contents in dry matter of shoot and root (B) of Zn-sensitive rice (*Oryza sativa* L.) cultivar grown hydroponically with either normal (0.15 µM) or high (2 mM) Zn without or with 1.5 mM Si for 72 h. Data are means ± S.D. of three replicates. Note:P-values indicate significance level based on two-way ANOVA. ^*^P<0.05, ^**^P<0.01. Data followed by different letters in the shoot or root are significantly different according to ANOVA (P <0.05).

The concentration of silicon in shoots and roots was decreased by 12.2% and 17.2%, respectively, in the Zn plus Si treatment compared with the corresponding Si treatment alone (Figure 3A).

Zn concentration in shoots and roots increased with increasing of Zn treatment concentration. The Zn concentration was much higher in roots than in shoots (Figure 3B). The ratio of Zn concentration in root/shoot was 3.0 under high Zn treatment, which, however, was increased by the addition of Si.

### Chlorophyll contents

**Figure 4:**
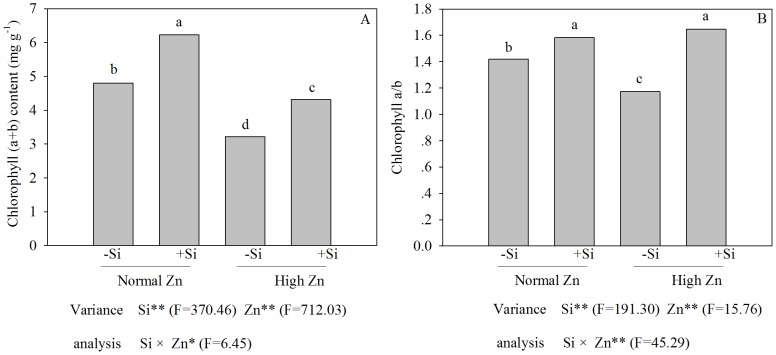
Chlorophyll (a+b) content (A) and the radio of Chlorophyll a/b(B) of Zn-sensitive rice (*Oryza sativa* L.) cultivar grown hydroponically with either normal (0.15 µM) or high (2 mM) Zn without or with 1.5 mM Si for seven days. Data are means ± S.D. of three replicates. Note:P-values indicate significance level based on two-way ANOVA. ^*^P<0.05, ^**^P<0.01. Data followed by different letters are significantly different according to ANOVA (P <0.05).

The contents of chlorophyll (a+b) and the value of chlorophyll a/b were decreased by 32.9% and 17.4%, respectively, in the presence of high Zn stress when compared with the control (*P*<0.05). The addition of Si to the plants treated with high levels of Zn led to 33.9% and 40.4% increases in the contents of chlorophyll (a+b) and the value of chlorophyll a/b, respectively, compared with levels in plants treated with Zn alone (Figure 4 A, B) (*P*<0.05).

### Chlorophyll fluorescence parameters

**Figure 5:**
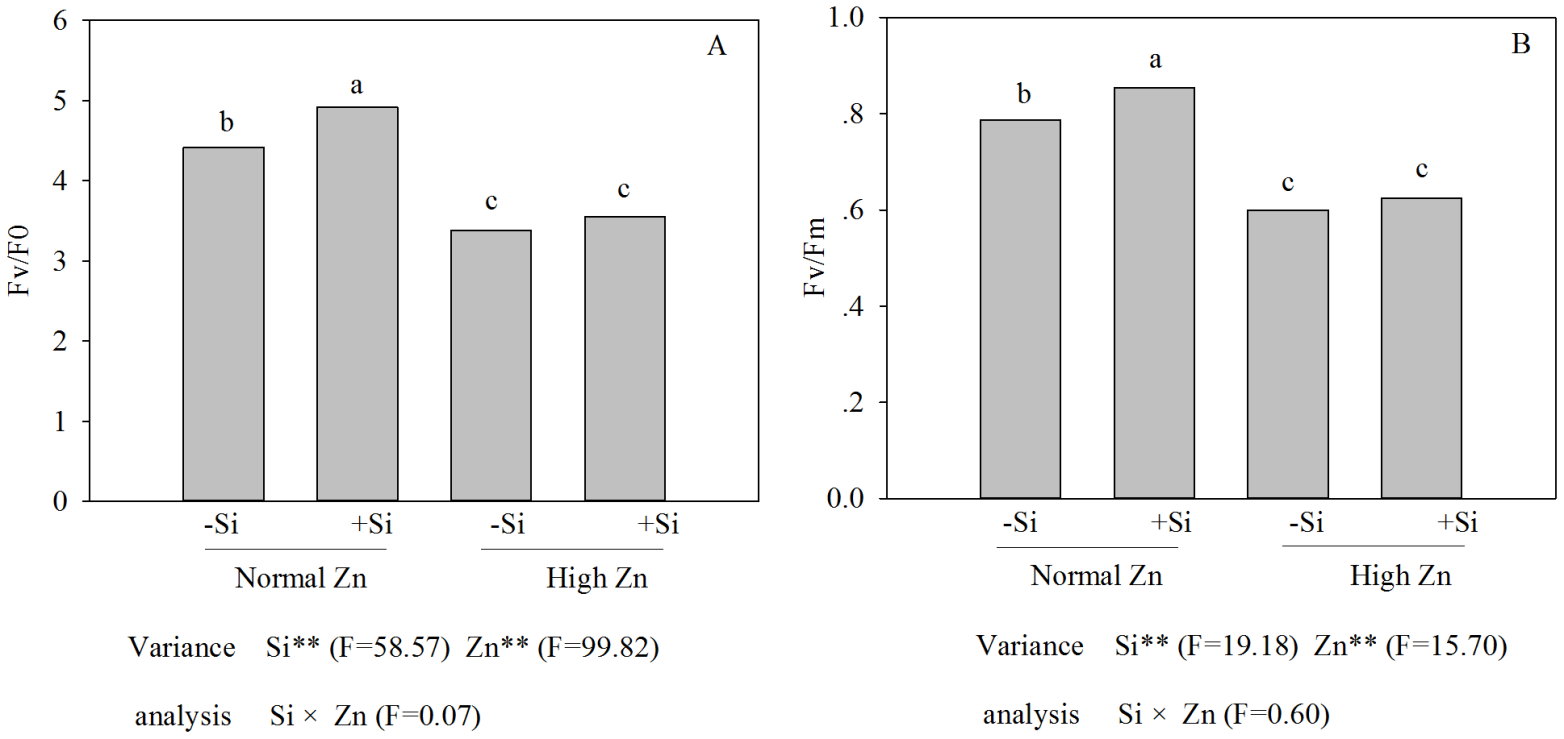
*F_v_/F_0_* (A) and *F_v_/F_m_* (B) of Zn-sensitive rice (*Oryza sativa* L.) cultivar grown hydroponically with either normal (0.15 µM) or high (2 mM) Zn without or with 1.5 mM Si for seven days. Data are means ± S.D. of three replicates. Note:P-values indicate significance level based on two-way ANOVA. ^*^P<0.05, ^**^P<0.01. Data followed by different letters are significantly different according to ANOVA (P <0.05).

Chlorophyll fluorescence was used as a non-invasive method to determine the functional state of the photosynthetic machinery. Compared with the control, the maximum yield of the photosystem II photochemical reactions (*F*_v_/*F*_m_) and the potential yield of the photosystem II photochemical reactions (*F*_v_/*F*_0_) were decreased under the high Zn treatment. The application of Si had no effects on *F*_v_/*F*_m_ and *F*_v_/*F*_0_ under high Zn stress, while under a normal Zn level, *F*_v_/*F*_m_ and *F*_v_/*F*_0_ were increased by the addition of Si (Figure 5).

### Photosynthesis parameters

**Figure 6:**
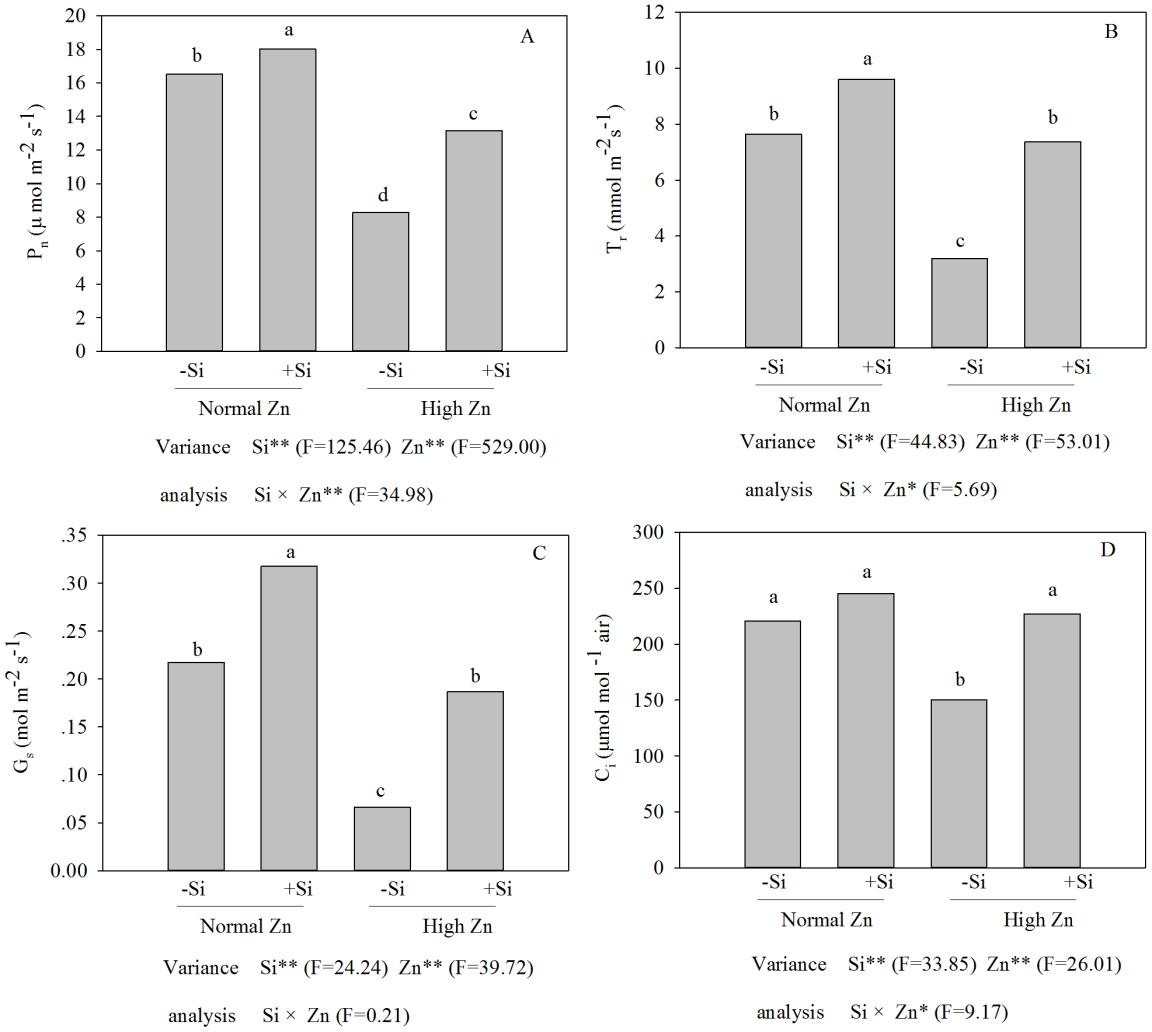
*P_n_* (A), *T_r_* (B), *G_s_* (C) and *C_i_* (D) of Zn-sensitive rice (*Oryza sativa* L.) cultivar grown hydroponically with either normal (0.15 µM) or high (2 mM) Zn without or with 1.5 mM Si for seven days. Data are means ± S.D. of three replicates. Note:P-values indicate significance level based on two-way ANOVA.^ *^P<0.05, ^**^P<0.01. Data followed by different letters are significantly different according to ANOVA (P <0.05).

The *P*_n_, *T*_r_, *G*_s_ and *C_i_* in plants receiving the high Zn treatment were reduced by 49.9%, 58.0%, 69.7% and 32.1%, respectively, compared with the corresponding control (Figure 6). However, compared with the values obtained from plants receiving the corresponding Zn treatment alone, the values of *P*_n_, *T*_r,_ *G*_s_ and *C*_i_ under the Zn plus Si treatment were increased. For example, the *P*_n_, *T*_r_, *G*_s_ and *C_i_* increased by 58.9%, 129.9%, 183.0% and 51.4%, respectively, under the Zn plus Si treatment compared with the corresponding Zn treatment alone.

### Chloroplast ultrastructure

**Figure 7:**
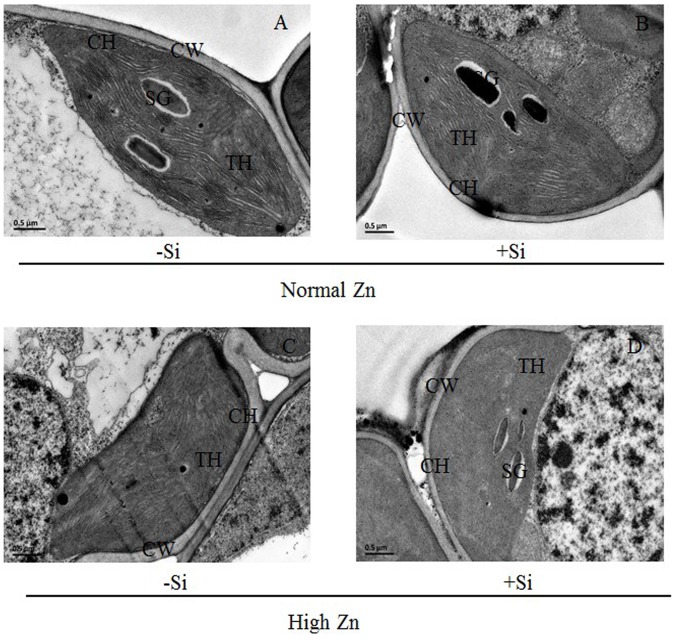
Chloroplast ultrastructure of Zn-sensitive rice (*Oryza sativa* L.) cultivar grown hydroponically with either normal (0.15 µM) or high (2 mM) Zn without or with 1.5 mM Si for seven days. CH = chloroplast, CW = cell wall, SG = starch grains, P = plastoglobuli, TH = thylakoid membranes, Vac = vacuole.

Under high Zn stress, the chloroplast's structure was observably swollen with chloroplast grana being destroyed (Figure 7C) as compared with the control (Figure 7A). This was considerably counteracted by the addition of Si (Figure 7D). The results showed that the addition of Si alleviated the negative effects of Zn stress on the chloroplast's ultrastructure in rice.

### Differential expression of genes

**Figure 8:**
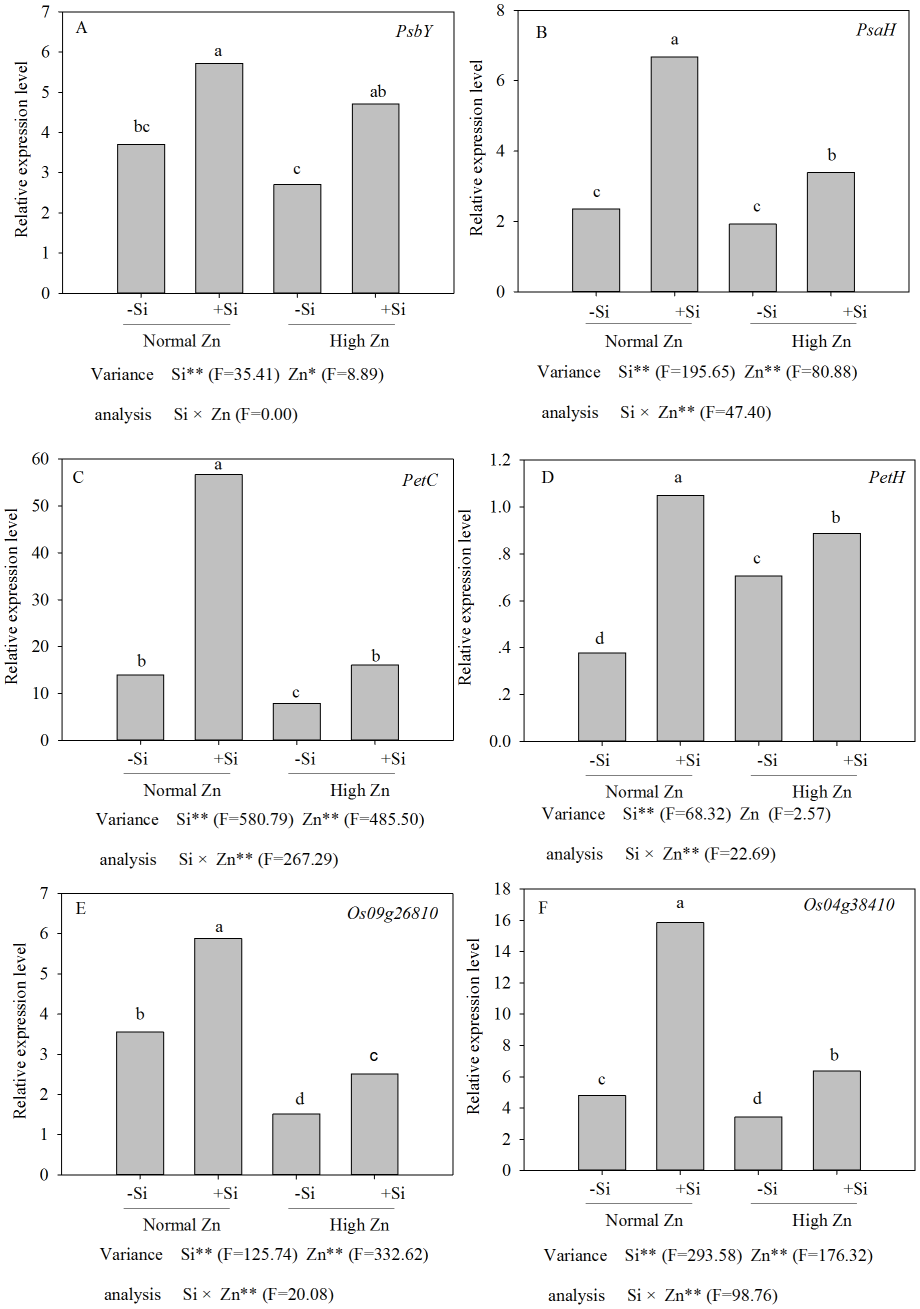
Genes expression of Zn-sensitive rice (*Oryza sativa* L.) cultivar grown hydroponically with either normal (0.15 µM) or high (2 mM) Zn without or with 1.5 mM Si for 72 h. Data are means ± S.D. of three replicates. Note:P-values indicate significance level based on two-way ANOVA. ^*^P<0.05, ^**^P<0.01. Data followed by different letters are significantly different according to ANOVA (P <0.05).

To investigate the beneficial effects of Si on Zn toxicity at the molecular level, we examined the expression of rice genes in response to Zn and Si treatments by real time RT-PCR. Interestingly, each gene showed a unique pattern of expression (Figure 8).

*PsbY* is a polyprotein of photosystem II (PSII). The expression level of the *PsbY* gene was down-regulated under high Zn stress but up-regulated by the addition of Si. Thus, the relative expression level in plants treated with Zn plus Si at 72 h was 1.7 times higher than that of plants treated with Zn alone. These results showed that the addition of Si could enhance the protein activity and improve the resistance to Zn stress (Figure 8A).

*PsaH* is a subunit of photosystem I (PSI) located in the center of the PSI dimer. The gene expression level of *PsaH* under high-Zn treatment was not different from that of the control. However, the addition of Si to the control or the Zn-treated plants increased the *PsaH* expression level at 72 h compared with the corresponding control plants or plants treated with high Zn alone. The expression level at 72 h was 2.8 times greater in the plants receiving Si treatment alone than in the control plants and 1.8 times greater in the plants receiving Si plus Zn treatment than in the plants receiving high-Zn treatment alone (Figure 8B).

The main function of the *PetC* gene is associated with the cytochrome's biological processes in photosynthesis. As shown in Figure 8C, *PetC* expression in rice was down-regulated under high-Zn stress at 72 h. Moreover, the expression levels at 72 h were higher in Si-treated plants than in Si-deprived plants under high-Zn stress (Figure 8C).

The relative expression level of *PetH* (*Os03g57120*) was up-regulated in rice cultivars under high-Zn stress compared with the control (Figure 8D). In addition, the expression level of the *PetH* gene was further increased by the addition of Si under high-Zn stress. However, the expression level was higher in Si-treated plants than in Si-deprived plants under high-Zn stress at 72 h.

Interestingly, the RNA level of the *Os09g26810* gene was even higher in plants treated with high Zn plus Si than in plants treated with high Zn alone (Figure 8E). For example, the RNA level of the *Os09g26810* gene at 72 h was 1.88 times higher in plants treated with Zn plus Si than in plants treated with high Zn alone.

There was a difference in *Os04g38410* expression levels between the Si-treated and Si-deprived plants under the normal Zn or the high-Zn treatment. The expression level at 72 h was 1.2 times higher in the presence of Si than in the absence of Si under the high-Zn stress (Figure 8F).

## Discussion

The concentration of soluble Zn in soils ranged from 4–270 µg L^−1^, however, in acidic soils, soluble concentrations of 7137 µg L^−1^ have been found, indicating that solubility is strongly, but inversely linked to soil pH [53]. The moderate Zn concentrations play an important role in several plant metabolic processes [54] and transcription of many genes [55]. However, excess Zn concentrations can cause cytotoxic effects (e.g, leaves become yellow), which were typically alleviated by addition of Si (Fig. 2A). In previous studies, the alleviation of Zn toxicity by application of Si has been observed in some plant species, including rice [40], [42]. These results indicated that Si could promote plant growth under high-Zn stress, and the present study supports this conclusion (Figure 2). The addition of Si increased the value of chlorophyll (a+b) content of rice plants treated with high Zn (Figure 4A); one of the reasons might be that addition of Si enhanced antioxidative defense capacity in tissues [42] and decreased the root-to-shoot transport of Zn (Figure 3). An additional reason can be attributed to the protective role of Si in photochemical reaction in photosynthesis, which could be explained by the change in chlorophyll fluorescence parameters in this study (Figure 5). The excessive accumulation of some heavy metals, such as Cu, Cd and Zn, may lead to changes of chlorophyll fluorescence parameters in leaves [56]. In the present study, the chlorophyll fluorescence parameters in rice leaves, *F*_v_/*F*_m_ and *F*_v_/*F*_0_, were inhibited by high-Zn stress. The ratio *F*_v_/*F*_m_ is proportional to the potential maximal quantum yield of PSII, and its reduction in rice leaves exposed to excess Zn might be attributed to loss of efficiency of PSII that is generally related to photoinhibition [57] or alteration in the levels of chlorophyll biosynthetic pathways or might be due to an altered stoichiometry between PSI and PSII, since this process was induced by an iron deficiency [58]. The effects of high Zn stress on the photosynthetic ability of rice were alleviated by the addition of Si, which might be linked to a protective mechanism that maintains the integrity of the photosynthetic machinery. A study by da Cunha and do Nascimento [59] showed that the effects of Si on alleviating Zn stress in maize depended greatly on its influence on structure. In the present experiment, a high Zn level induced a chlorophyll deficiency (Figure 4), which was closely correlated with a decreasing *P*_n_ (Figure 6). Similar results have been observed in ryegrass and rapeseed [43], [60]. Furthermore, the ultrastructure of the chloroplasts was greatly changed and exhibited disturbed shapes under the high-Zn stress (Figure 7). These results were in accordance with the Zn-treated maize [61]. *P*_n_, *T*_r,_ *G*_s_ and *C*_i_ were decreased in Zn-treated rice plants as compared with the control (Figure 6). Therefore, the high-Zn-induced inhibition of photosynthetic processes in rice was due to stomatal restriction, which might be caused by the inhibition of key enzyme activities in the Calvin cycle, the photosynthetic electron transport chain and Rubisco activity [62], as well as by the damage of the chloroplasts' ultrastructure [53]. However, Detmann et al. [63] and Sanglard et al. [64] proposed a role for Si in improving photosynthesis through decreased diffusive limitations. These research results show that Si-increased mesophyll conductance was more prominent than Si-increased stomatal conductance and the protection of chloroplasts by Si might play an important role in increasing rice tolerance to Zn stress.

Photosynthesis is one of the most Zn-sensitive plant processes, with Zn adversely affecting the photosynthetic electron transport chain. This effect could be due to metal-induced reductions in the levels of photosynthetic pigments, the inhibition of the formation or disintegration of PSI, PSII and light-harvesting pigment-protein complexes, or effects on the intermediate electron carrier system-Cyt b6/f complexes linking the two optical systems of PSI and PSII [65]. However, the addition of Si increased the ratio of chlorophyll a/b and enhanced electron transport chain of plants under high-Zn stress (Figure 4B). To investigate the beneficial effects of Si on Zn toxicity at the molecular level, we examined the expression of rice genes in response to Zn and Si treatments by real time RT-PCR. Interestingly, each gene showed a unique pattern of expression (Figure 8). *PsbY* is one of the low-molecular-mass subunits of oxygen-evolving PSII. Isolated photoactive PSII complexes may contain up to 22 polypeptides [66], [67], and many have been suggested to constitute the oxygen-evolving complex. Previous studies have shown that in the absence of *PsbY*, the mutant did not affect the composition of PSII and the growth of photoautotrophic algae [68]. However, tests proved that *PsbY* was a manganese-binding polypetide with L-arginine metabolizing enzyme activity, pointing to the possible role of this subunit in the function of the Mn_4_Ca-cluster in oxygen-evolving PSII [69], [70], [71]. In this study, the relative gene expression level of *PsbY* increased with the addition of Si under high-Zn stress (Figure 8A), suggesting that the manganese-binding capacity was increased, and water oxidation was enhanced in PSII of rice. These results showed that the addition of Si could induce the rapid expression of the *PsbY* gene in rice, a gene that has a novel manganese-binding, low-molecular-mass protein associated with PSII. The stimulation of *PsbY* mRNA transcripts might increase the activity of PSII and the electron transfer rate in rice, which was observed by an increase in chlorophyll content. *PsaH* is one of the key genes that encode the 13 polypeptides and is a membrane protein with a 10 kD molecular weight that is encoded by nuclear genes. It lies at the surface of PSI and contacts the *PsaA* and *PsaD* genes [72]. Naver's studies [73] showed that PSI-H played an important role in maintaining the stability of the photosynthetic co8y. Lunde et al. [74] found that the lack of the PSI-H subunit resulted in complex II (LCH II) not being able to transfer energy to system I, which led to a transition delay from photosynthetic state I to state II. In our experiments, the expression level of *PsaH* was unchanged after high-Zn exposure for 72 h in the absence of Si, which was, however, rapidly raised in the presence of Si (Figure 8B). The addition of Si was conducive to enhancing *PsaH* expression level, consequently improving the capacity of photosystem I.

The *PetC* gene encodes the polypeptide binding the Rieske FeS center of the cytochrome *bf* complex. The protein is strongly associated with the cytochrome *bf* complex on the lumenal side of the thylakoid membrane, although it does not appear to be an intrinsic membrane protein [75]. The main function of the *PetC* gene is involved in the cytochrome's biological processes in photosynthesis. As shown in Figure 8C, the *PetC* gene's expression level was down-regulated under high-Zn stress compared with the control. These results indicated that high-Zn stress affected the biological processes of the cytochrome, and resulted in a decrease in the chlorophyll a and b contents. However, the addition of Si increased the *PetC* gene's rapid expression, protected the cytochrome in the rice leaves and alleviated the destructive effects of high Zn on the chloroplasts' structure (Figure 7).

*PetH* gene encoding ferredoxin NADP+ reductase catalyzes NADP+ to generate NADPH at the end of the photosynthetic electron transport chain, maintaining the content of reducible glutathione in the cells. The *PetH* relative expression level increased under high-Zn stress, suggesting that the reactive oxygen species produced in rice plants under high-Zn stress could be quickly and effectively removed. The relative gene expression level of *PetH* was further increased by the addition of Si under high-Zn stress (Figure 8D). These results were in accordance with the ratio of chlorophyll a/b which reflected how much energy was used by plants. This may be related to the alleviation caused by Si.

Light harvesting complex II (LHCII) structure plays a crucial role in photosynthesis. Multiple genes encoding subunits of the LHCII complex are adapted to changes in the external environmental conditions by changing the composition of the structure. The expression levels of *Os09g26810* and *Os04g38410* genes decreased under high-Zn stress and increased with the addition of Si. While the decreasing and increasing trends were the same, the expression levels were higher with Si than without Si. These results indicate that the expression levels of the two genes were inhibited after a certain time under the high-Zn treatment, and were up-regulated by the addition of Si to protect from inhibition by high-Zn stress (Figure 8E, F).

## Conclusions

High Zn stress inhibited the growth of rice seedling, and destroyed the structure and function of the photosystem. The addition of Si could protect the photosynthetic pigments in leaves, alleviate the damage to chloroplast ultrastructure, and increase the expression of genes associated with photosynthesis. This consequently alleviated the high-Zn-induced damage to photosynthesis, and thus, enhanced the ability of plants to tolerate high-Zn stress. Taken together, all these results suggest that Si-mediated alleviation of Zn toxicity to plant growth and photosynthesisis was mainly attributed to Si-reduced root-to-shoot Zn translocation, and the Si-regulated expression of genes related to photosynthesis.
